# Integrative Analysis of Transcriptome and Metabolome Reveals Salt Stress Orchestrating the Accumulation of Specialized Metabolites in *Lycium barbarum* L. Fruit

**DOI:** 10.3390/ijms22094414

**Published:** 2021-04-23

**Authors:** Shuang Lin, Shaohua Zeng, Biao A, Xiaoman Yang, Tianshun Yang, Guoqi Zheng, Guilian Mao, Ying Wang

**Affiliations:** 1 Key Laboratory of South China Agricultural Plant Molecular Analysis and Genetic Improvement, Guangdong Provincial Key Laboratory of Applied Botany, South China Botanical Garden, Chinese Academy of Sciences, Guangzhou 510650, China; linshuang@scbg.ac.cn (S.L.); Abiao@scbg.ac.cn (B.A); yangxm@scbg.ac.cn (X.Y.); tianshun1982@126.com (T.Y.); 2 College of Life Sciences, Gannan Normal University, Ganzhou 341000, China; 3 Center of Economic Botany, Core Botanical Gardens, Chinese Academy of Sciences, Guangzhou 510650, China; 4 University of Chinese Academy of Sciences, Beijing 100049, China; 5 School of Life Sciences, Ningxia University, Yinchuan 750021, China; 13995011126@163.com (G.Z.); fransis-0327@163.com (G.M.)

**Keywords:** *Lycium barbarum* L., salt stress, fruit quality, carotenoids, flavonoids

## Abstract

Salt stress seriously affects yield and quality of crops. The fruit of *Lycium barbarum* (LBF) is extensively used as functional food due to its rich nutrient components. It remains unclear how salt stress influences the quality of LBF. In this study, we identified 71 differentially accumulated metabolites (DAMs) and 1396 differentially expressed genes (DEGs) among ripe LBF with and without 300 mM of NaCl treatment. Pearson correlation analysis indicated that the metabolomic changes caused by salt stress were strongly related to oxidoreductases; hydrolases; and modifying enzymes, in particular, acyltransferases, methyltransferases and glycosyltransferases. Further analysis revealed that salt stress facilitated flavonoid glycosylation and carotenoid esterification by boosting the expression of structural genes in the biosynthetic pathways. These results suggested that salt stress prompts the modification of flavonoids and carotenoids to alleviate ROS damage, which in turn improves the quality of LBF. Our results lay a solid foundation for uncovering the underlying molecular mechanism of salt stress orchestrating LBF quality, and the candidate genes identified will be a valuable gene resource for genetic improvement of *L. barbarum*.

## 1. Introduction

Soil salinity is a severe land degradation attributed to natural ecological elements and anthropogenic activities. It is estimated that more than 20% of all irrigated lands are affected by excess salt concentrations, and nearly 2000 ha of arable land is being lost every day by salinization processes [1]. Cultivated land has been restricted by salt stress for a long time, and the problem will exacerbate in many regions in the world due to climate change or manmade damage.

Salinity has deleterious effect on seed germination, plant growth and crop productivity. Yield decrease caused by excessive salt has been found in many cereals, such as rice, wheat, barley and maize [2]. Furthermore, salt stress induced improper development of grains, thereby altering the composition and quality of cereal grains [3]. The quality of grains was affected by salt stress, including marked reductions in amylose starch contents in rice [4] and the substantial decline in protein and starch contents in wheat [5]. Thus, salt stress imposes negative effects on the yield and quality of cereals.

In addition, recent studies documented that salt stress also affects the fruit quality, resulting in changes of metabolite accumulation in fruits. Without significantly affecting fruit yield, mild salt stress improved strawberry fruit quality by increasing the content of sucrose, anthocyanins and antioxidant compounds. Salt stress controlling fruit quality can also be found in Solanaceae fruits. In tomato fruits, salt stress in some cases led to a 2–3-fold increase in the lycopene content [6], and also improved the accumulation of starch, sucrose and amino acids [7,8]. The content of capsaicinoids in chili pepper increased by 389% at 60 mM of NaCl treatment [9]. Taken together, these studies indicated that salt treatment largely changes the content and/or proportion of nutrient components in fruits, which further orchestrates the fruit quality properties.

Intriguingly, the fluctuation of metabolites regulated by salt signals is a way to help fruits gain stress tolerance by maintaining redox hemostasis and mitigating impairment. In *Solanum nigrum* L, salt stress induced expression of several key genes in carotenoid and flavonoid biosynthetic pathways to enhance accumulation of *β*-carotene, lutein, and quercetin 3-*β*-D-glucoside, showing a great extent of antioxidant activity [10]. There is a large consensus that flavonoids function as ROS scavengers for inhibiting ROS generation and reducing ROS effects [11]. Polar carotenoids present in the lipid membrane have been shown to limit molecular oxygen penetration into the lipid bilayer [12]. Since carotenoids and flavonoids can prevent and scavenge ROS, they refer to antioxidant compounds for the plant internally, as well as functional components for humans who intake these metabolites in their daily diet. Hence, the level of these functional components is an important factor determining the quality of crops and fruits.

The *Lycium barbarum* L. fruit (LBF) is also known as the Goji berry. There are various ways to consume LBF, such as eating it raw or drinking it as an ingredient of juice, wine or tea [13]. People all over the world, especially in east Asia, regard LBF as a functional food because its’ extracts are beneficial for age-related disorders, immune disorders, neuromodulation and so on [14]. Phytochemistry studies confirmed that carotenoids and flavonoids are major bioactive components of the Goji berry [13]. Given that *L. barbarum* is mainly grown in northwest China, where there is serious soil salinity, and the photosynthesis of *L. barbarum* seedling was proved to be greatly interfered with by salt stress [15], we are interested in determining whether the stress signal would affect the accumulation of bioactive constituents in LBF.

In this study, physiological, transcriptomic and metabolomic investigations were conducted in LBF with and without 300 mM of NaCl treatment. Our results indicate that enzyme and non-enzyme systems were activated to detoxify the ROS triggered by salt stress. Several classes of metabolic biosynthesis were up-regulated in both transcriptional and metabolic levels, especially for esterified carotenoids and flavonoid glycosides. The fact that salt stress treatment improved the quality of LBF is extensively discussed.

## 2. Materials and Methods

### 2.1. Plant Materials and Treatments

The salt treatment experiments were carried out in Zhongning County of the Ningxia Hui Autonomous Region, China, in July 2019. Our primary experiment indicated that the concentration of fruit carotenoid in the *Lycium barbarum* plant irrigated with 300 mM of NaCl once a week was higher than that treated with 200 mM of NaCl, which is defined as the critical concentration of the halophytic plant. Consequently, 300 mM of NaCl was utilized to treat the plants in this study. From April to September, the frequency of irrigation was reduced to once a fortnight. After three months of treatments, fruits of two developmental stages, namely S1 (green stage) and S4 (ripe stage), were harvested according to our previous study [16]. Each group of more than thirty fruits was randomly collected and randomly separated into four replicate groups, frozen in liquid nitrogen and kept at −80 °C for later use.

### 2.2. Measurement of Physiological Indexes

The contents of oxygen free radical (OFR), hydrogen peroxide (H_2_O_2_) and malondialdehyde (MDA) were measured by assay kits based on the hydroxylamine hydrochloride method, titanium sulfate colorimetric method and thiobarbitoric acid (TBA) method, respectively. The activities of superoxide dismutase (SOD), peroxidase (POD) and catalase (CAT) were determined by the nitroblue tetrazolium (NBT) method, guaiacol-H_2_O_2_ reaction and H_2_O_2_ scavenge assay, respectively. Diazo blue B was used to detect ascorbic acid (AsA), and BCA based on the biuret reaction was adopted for the soluble protein measurement. The acid ninhydrin method was used for the estimation of proline content, while the reaction of Fe^3+^-TPTZ at 593 nm indicated the total antioxidant capacity (T-AOC). All of these measurements were conducted using assay kits purchased from Suzhou Comin Biotechnology Company (http://www.cominbio.com/index.html) on 16 October 2020.

To extract total flavonoids, 1.5 g of fresh fruit powder was subjected to ultrasonic treatment in 2 mL of methanol for 30 min. The content of total flavonoids was estimated based on the aluminum chloride colorimetric method. To 0.5 mL of extract, 0.3 mL of 5% NaNO_2_ (mixed well) was added and left to stand for 5 min at room temperature. Then, 0.3 mL of Al(NO_3_)_3_ was added for further reaction. After 6 min, 2 mL of 1 M NaOH was added into the mixture, and then the OD_510_ was measured with a photospectrometer. Rutin stock solution was diluted into five concentrations to make a standard calibration curve. Quantitative analysis of total flavonoids was performed based on the rutin standard curve: y = 1.1455X − 0.0176, R^2^ = 0.9977 (y and X denote flavonoid relative peak area and concentration, respectively).

The carotenoids were extracted as described previously [16] with modifications. Specifically, frozen fruit powder of 200 mg was subjected to ultrasonic extraction of carotenoids for 30 min in a 3 mL mix reagent of hexane/acetone/ethanol (2:1:1, *v*/*v*/*v*; with 0.1% butylated hydroxytoluene) twice. The supernatant was combined with 3 mL of saturated NaCl solution, shaken for 1 min, and the clear supernatant was moved to a new tube after standing stratification. The residue was partitioned with 3 mL of hexane twice, and all of the supernatants were combined and dried in a rotary vacuum evaporation system. Two milliliters of dichloromethane was used to re-dissolve the carotenoid extracts, and the OD_450_ was measured. The content of total carotenoids was calculated from a calibration curve constructed by zeaxanthin with five concentration grades: y = 49.628X + 0.0234, R^2^ = 0.9986 (y and X denote carotenoid relative peak area and concentration, respectively).

### 2.3. Transcriptome Sequencing Analysis

The total RNA was extracted from frozen fruit, and the mRNA libraries of samples were constructed and sequenced with the Illumina Hiseq 4000 platform. The quality control and data processing were conducted by the QoRTs software package [17], and clean reads were then mapped to the *Lycium ruthenicum* M. genome using HISAT2. Based on the raw count data, differential expression analysis between samples was performed by the edgeR software [18]. Genes satisfying |log2FoldChange| ≥ 1 and *p* value < 0.05 were defined as differentially expression genes (DEGs) and subjected to Gene Ontology (GO) and KEGG enrichment analysis through clusterProfiler [19].

### 2.4. Untargeted Metabolomic Analysis

Freeze-dried fruit powder of 100 mg was extracted at 4 °C overnight with 0.6 mL of 70% aqueous methanol followed by centrifugation at 10,000× *g* for 10 min. The extracts were absorbed and filtrated.

The sample extracts were analyzed using a UPLC-ESI-MS/MS system (UPLC, SHIMADZU CBM30A system; MS, Applied Biosystems 4500 Q TRAP) equipped with a Waters ACQUITY UPLC HSS T3 C18 column (1.8 µm, 2.1 mm × 100 mm). The column oven was set to 40 °C, and the injection volume was 4 μL. The mobile phase consisted of solvent A, pure water with 0.04% acetic acid, and solvent B, acetonitrile with 0.04% acetic acid. A gradient program was employed as follows: 0–10 min, 5% B; 10–11 min, 5–95% B; 11–14 min, 95–5% B.

The MS System was equipped with an ESI Turbo Ion-Spray interface, and the operation parameters were as follows: ion source, turbo spray; source temperature 550 °C; ion spray voltage (IS) 5500 V (positive ion mode)/−4500 V (negative ion mode); ion source gas I (GSI), gas II (GSII) and curtain gas (CUR) were set at 50, 60 and 30 psi, respectively; the collision gas (CAD) was high.

Unsupervised principal component analysis (PCA) was performed by statistics function prcomp within R. The data were unit variance scaled before unsupervised PCA. Differentially accumulated metabolites (DAMs) between groups were determined by VIP ≥ 1 and FC (fold change) > 1.5 or FC < 0.67. VIP values were extracted from the OPLS-DA result, which was generated by using the R package MetaboAnalystR [20]. The data were log-transformed (log2) and mean-centered before OPLS-DA. In order to avoid overfitting, a permutation test (200 permutations) was performed.

Identified metabolites were annotated using the KEGG compound database, and annotated metabolites were then mapped to the KEGG pathway database.

### 2.5. Carotenoids Quantitative Analysis

The carotenoid extraction method was as mentioned in 2.1. After drying the extract in the rotary vacuum evaporation system, 800 μL dichloromethane was used to dissolute carotenoids, and contaminants were removed by syringe filtering with 0.2 μm of polytetrafluoroethylene membrane. The instruments used in this section were made of glass for corrosion avoidance, in particular, the extraction tubes, pipettes and reagent containers.

The carotenoids analysis was performed by using a LC-2030C 3D liquid chromatographer (SHIMADZU) with a reverse-phase C_30_ carotenoid column (250 × 4.6 mml.D. S-5 μm, YMC). The column was operated at 30 °C and the flow rate was set at 1 mL/min. Methanol/acetonitrile (3:8, *v*/*v*) (A) and dichloromethane/hexane (1:1, *v*/*v*) (B) contributed to the binary mobile phase and eluted as follows: 0–2.5 min, 10% B; 2.5–4.5 min, 10–30% B; 4.5–25.5 min, 30–50% B; 25.5–27.5 min, 50–10% B; and 27.5–30 min, 10% B.

Standards, including *β*-carotene, *β*-cryptoxanthin, lycopene, zeaxanthin and zeaxanthin dipalmitate, were used to identify carotenoids in the extracts. The relative contents of carotenoid components were determined by the peak area at 450 nm.

### 2.6. Quantitative Real-Time PCR Analysis

Quantitative real-time PCR analysis was conducted to validate the accuracy of transcriptome. A total of 15 candidate genes were selected for this experiment, and *Actin I* was used as the reference gene. Total RNA was extracted from samples and the MonAmp^TM^ ChemoHS qPCR Mix was used as a PCR reagent. The PCR was performed in a Roche LightCycle 480 system, and the program was set as follows: 95 °C, 10 min; 95 °C for 10 s; and 60 °C for 30 s, 40 cycles. Data analysis was performed using the 2^−ΔΔCt^ method.

### 2.7. Statistical Analysis

The results of the physiological analyses were presented as mean ± standard deviation with at least three biological replicates. Statistical analysis was carried out by comparing the average of each group by two-way analysis of variance (ANOVA), according to Sidak’s multiple way test at *p* < 0.05.

The integrated analysis of metabolome and transcriptome was conducted by calculating the Pearson correlations between DEGs and DAMs. Pairwise relations with *p* value < 0.05 and |PCC| > 0.9 were used to construct the network and were visualized with the Cytoscape software (version 3.8.0).

## 3. Results and Discussion

### 3.1. Physiological Response of LBF under Salt Stress

**Figure 1:**
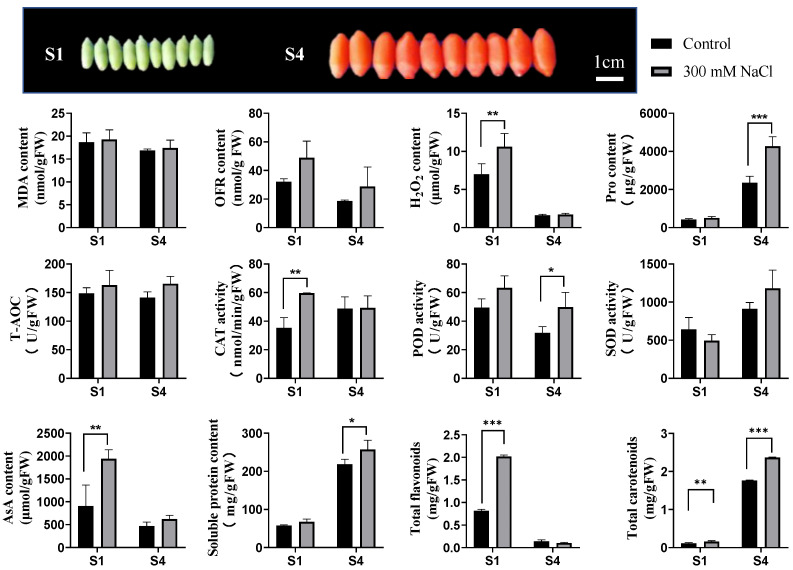
Physiological response of fruits from *Lycium barbarum* treated with 300 mM of NaCl for three months. Reactive oxygen species (ROS) and oxidation products (including oxygen free radical (OFR), hydrogen peroxide (H_2_O_2_) and malondialdehyde (MDA)), antioxidant enzymes activities (including superoxide dismutase (SOD), peroxidase (POD) and catalase (CAT)), non-enzymatic antioxidant ascorbic acid (AsA) and total antioxidant capacity (T-AOC) were detected as described in the Materials and Methods. In addition, osmoprotectant proline (Pro) and soluble protein content were also measured. Data presented as mean ± SD, *n* = 3. Symbols *, ** and *** represent the significance differences at *p* < 0.05, *p* < 0.01 and *p* < 0.001 level, respectively.

To investigate the effects of salt stress on LBF, several physiological parameters in S1 (green stage) and S4 (ripe stage) fruits were detected. As shown in Figure 1, the level of MDA in control fruits (CKS1 and CKS4) had no significant difference compared to salt treatment samples (STS1 and STS4), indicating that lipid peroxidation did not cause remarkable damage in LBF. The content of oxygen free radical (OFR), the enzyme activity of SOD and the total antioxidant capacity (T-AOC) in S1 and S4 samples showed no statistical differences between control and salt treatment. The content of H_2_O_2_ showed significant increase in STS1 when compared to CKS1, and no difference was observed between STS4 and CKS4. The enzymatic activity of CAT decomposing H_2_O_2_ was up-regulated in STS1 but showed no changes in S4 fruit. The content of H_2_O_2_ in tested samples had a similar pattern to that of CAT. However, the enzymatic activity of POD in samples only increased in STS4.

As the major constituent of LBF free amino acids, proline (Pro) content was markedly increased in the S4 fruit, indicating that Pro is one of the most important antioxidants for S4 fruit tolerance to salt stress. A previous study documented that enhanced accumulation of Pro improved plant tolerance to various abiotic stresses by alleviating cytoplasmic acidosis and maintaining NADP^+^: NADPH balance [21].

Ascorbic acid (AsA) is considered the most powerful ROS scavenger because of its ability to donate electrons in a number of enzymatic and non-enzymatic reactions [22]. The content of AsA in S1 fruit was sharply increased but not in S4 fruit under salt stress. AsA was reported to be abundantly accumulated in mature leaves with fully developed chloroplast and chlorophyll [23]. The absence of chloroplast and chlorophyll in S4 fruits might be one of the reasons for S4 fruits with less AsA under salt stress.

Additionally, the level of soluble protein was monitored, which contained a series of enzymes serving in antioxidant metabolite biosynthesis, such as carotenoids, flavonoids and glycine betaine. As soluble protein content was up-regulated in ripe fruits, salt signaling might have stirred a catalytic reaction, either a primary or secondary metabolism.

Flavonoids and carotenoids are main secondary metabolites in LBF, which function as low molecular antioxidants. The total flavonoid content was only remarkably increased in S1 fruit under 300 mM of NaCl treatment. Nevertheless, the total carotenoids were significantly up-regulated by salt stress both in S1 and S4.

Collectively, these results indicated that LBF adopted different strategies to mitigate ROS level at different developmental stages when suffering salt stress. Namely, CAT, AsA and flavonoids contributed to mitigating ROS in S1 fruit while POD, Pro and carotenoids contributed to this in S4 fruits.

### 3.2. Non-Targeted and Targeted Metabolomic Analysis of CKS4 vs. STS4

To illustrate metabolomic change in ripe LBF, untargeted metabolome was profiled for control and salt treatment samples using HPLC-ESI-MS/MS. The representative total ion chromatograms (TIC) are shown in Figure S1. In metabolite ion features, a total of 391 metabolites were tentatively identified to biochemical pathways. The detailed information of these known metabolites in ripe fruit samples were completely listed in Table S1. Among annotated metabolites, phenolic acids were the predominant component (16.62%), successively followed by flavonoids (16.37%), alkaloids (13.30%), lipids (12.79%), amino acids and their derivatives (12.53%) and others (10.23%).

The results of the pairwise comparison performed through the OPLS-DA approach showed a clear cluster separation between CKS4 and STS4 (R^2^X = 0.482, R^2^Y = 0.998, Q^2^ = 0.87), indicating a robust difference in metabolomic profiles between S4 fruits with and without treatment (Figure S2A).

**Figure 2:**
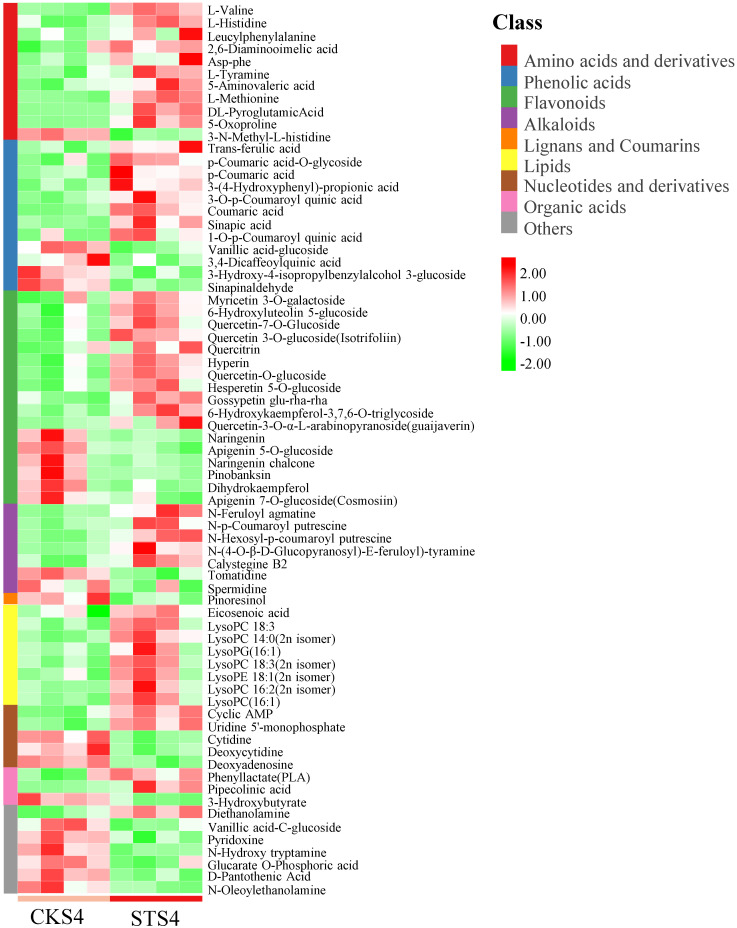
The profile of differentially accumulated metabolites (DAMs) in *Lycium barbarum* fruit. S4 fruit indicate that fruit was collected at ripe stage. ST and CK refer to fruit treated with and without 300 mM of NaCl, respectively. Metabolites with VIP > 1 and fold change >1.5 or <0.67 were defined as DAMs. The color key indicates the relative content of metabolite of low to high levels from green to red.

There was a total of 71 metabolites defined as differentially accumulated metabolites (DAMs) (VIP > 1) in non-targeted metabolome data (Figure S2B). These DAMs mainly belong to four classes, flavonoids (17), phenolic acids (12), amino acids and derivatives (11) and lipids (8). A comparison of the relative content of each DAM indicated that most of these metabolites were prompted by salt treatment (Figure 2).

Considering the fact that non-targeted metabolome cannot detect lipid-dissolved compounds, we performed HPLC to detect carotenoids in S4 fruit (Figures S3 and S4). As shown in Figure S4, zeaxanthin was significantly reduced, while *β*-carotene was increased in STS4 samples compared to CKS4 samples. As the most abundant carotenoid in LBF, zeaxanthin dipalmitate (ZDP) content was significantly increased in STS4 fruit, which may be responsible for the reduction of zeaxanthin. Other unidentified carotenoid esters were assigned as CE1-8. Among them, CE2, CE3, CE5, CE6 and CE8 were significantly up-regulated by salt treatment.

Taken together, our results indicated that salt stress remarkably promoted S4 LBF to accumulate the most DAMs, including phenolic acids, flavonoids, and carotenoids. Since some of them function as nutrient components, a preliminary conclusion was drawn that salt stress improves fruit quality of *L. barbarum*.

### 3.3. Transcriptomic Analysis

Considering the fact that salt stress remarkably enhanced the content of nutrient components, the effects of salt stress on orchestrating the transcription regulation in S1 and S4 fruits were monitored. In this study, an average of 47,865,017 clean reads were generated per sample representing 7,134,374,834 nucleotides (Table S2). GO annotation was applied to describe the biological functions of transcripts (Figure S5), and qRT-PCR was conducted to confirm the accuracy of transcriptome profiling. As shown in Figure S6, similar expression patterns were found between RPKM value and relative expression level, indicating that the data of RNA-Seq were valid and reliable.

In the PCA plot (Figure S7A), the first principal component (PC1) accounted for up to 55.18% of variability, separating S1 and S4 samples clearly; the second principal component (PC2) accounted for 6.46% of variability, dividing S4 samples with or without treatment but not S1 samples. Furthermore, pairwise comparisons were performed for the identification of differentially expressed genes (DEGs). STS1 had 621 DEGs with 401 down-regulated and 220 up-regulated when compared to CKS1. STS4 had 1,396 DEGs with 999/397 up- and down-regulated genes, respectively (Figure S7B). It seemed that S4 fruit was more sensitive to salt stress than S1 fruit.

GO enrichment analysis of DEGs showed that a series of oxidation-reduction relevant terms were ranked in the top 10 enriched terms among the comparison between CKS1 and STS1, with none of them being metabolic relevant (Figure S8A). However, the enriched GO terms among S4 samples, “Catalytic activity” and “Metabolic process” containing over 500 transcripts in each group, indicated that salt stress does briskly affect metabolic processes in ripe fruits.

As the metabolism process was susceptible to salt treatment in ripe fruits, DEGs in S4 samples, either down- or up-regulated genes, were performed with KEGG enrichment analysis. The down-regulated DEG clusters were enriched in “Carotenoid biosynthesis”, “Vitamin B6 metabolism” and “Nitrogen metabolism” (Figure S8B). In the up-regulated DEG clusters, “Metabolic pathways” were enriched supporting the GO enrichment result. The enriched pathways included lipid metabolism-involved pathways (for instance, “Biosynthesis of unsaturated fatty acids”, “alpha-Linolenic acid metabolism” and “Fatty acid metabolism”), and secondary metabolism pathways (such as “Phenylpropanoid biosynthesis”, “Diterpenoid biosynthesis”, “Stilbenoid, diarylheptanoid and gingerol biosynthesis” and “Flavonoid biosynthesis”).

In summary, the transcriptomic analysis results suggested that salt stress affected S4 fruit more than S1 fruit, especially the expression of genes assigned to metabolic processes and enriched in pathways belonging to metabolism.

### 3.4. Integrative Analysis of Transcriptome and Metabolome

To globally understand the regulation network in S4 fruit, the transcriptomic and metabolomic analysis were integrated. We combined 71 DAMs in non-targeted metabolome with eight differentially accumulated carotenoids (Figure 2 and Figure S4) to construct the DAMs pool. The Pearson correlation coefficient (PCC) of the total 79 DAMs and 1396 DEGs were calculated. As shown in Figure S9, the DEGs, which were shown to be strongly related to DAMs with |PCC| > 0.9 and *p* < 0.05, could be categorized into five modules. Module I was a cluster of oxidoreductases, such as cytochrome P450, dehydrogenase, FAD/NAD(P) binding oxidase, haem peroxidase (HPX) and glutathione S-transferase (GST). GST is one of the key components in the Ascorbate-Glutathione (AsA-GSH) pathway, participating in ROS detoxification [24]. Module II contained DEGs encoding a series of modifing enzymes, including acetyltransferases, glycosyl transferases, aminotransferases and SAM-dependent methyltransferases (MTase). Recently, a novel *MTase1* gene was isolated from salt-tolerant sweet potato and characterized functionally as a salt tolerance enhancer by regulating osmotic balance, protecting membrane integrity and increasing ROS scavenging capacity [25]. Module III was a large class of hydrolases. The three largest sub-families in this cluster were alpha/beta hydrolases, glycoside hydrolases and nucleoside phosphate hydrolases, followed by various esterases, in particular, phosphoesterases, pectinesterases, acetylesterases, PC-esterases, GDSL lipases/esterases and fatty acid hydrolases. Module IV contained MYB, bHLH, WD40, AP2/ERF and proteins carried zinc fingers domain. It is well studied that multiple MYB, WD40 and bHLH form MBW complexes together, which positively or negatively regulate phenolic acid and flavonoid biosynthesis [26]. Module V and VI were serine/threonine protein kinases, ATPases and peptidases.

To sum up, the biosynthesis and modification of STS4 DAMs were closely related to the transcription factors and proteins involved in oxidoreductases, transferases and hydrolases.

### 3.5. Fatty Acid and Lipid Metabolisms

The KEGG enrichment analysis revealed that biosynthesis of unsaturated fatty acids and alpha-linolenic acid and fatty acid metabolisms were ranked in the TOP 10 pathways based on the up-regulated DEGs in STS4 vs. CKS4 (Figure S8B). As shown in Figure 2, there was a cluster of lysolecithins defined as DAMs, which attracted our attention, illustrating how salt stress affected fatty acid and lipid metabolisms in S4 LBF.

**Figure 3:**
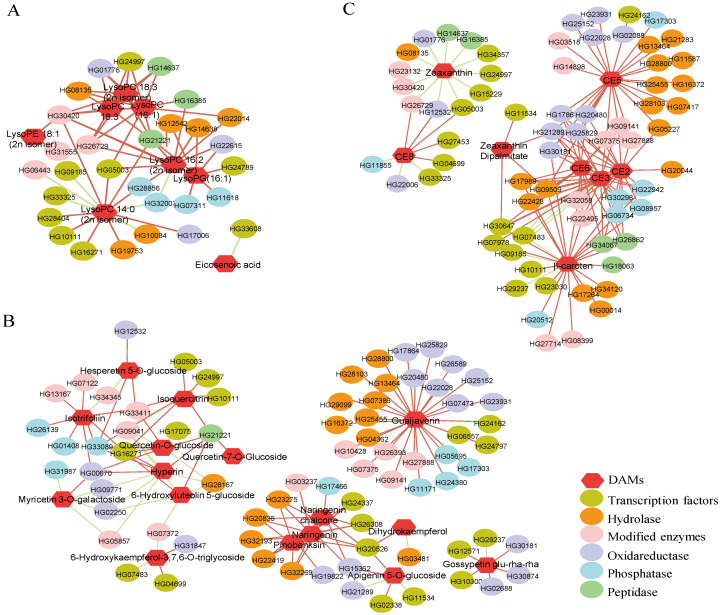
The co-expression network of differentially expressed genes (DEGs) and accumulated metabolites (DAMs) involved in lipid (**A**), flavonoid (**B**) and carotenoid (**C**) pathways. The red hexagons represent differentially accumulated lipids, flavonoids and carotenoids; the ellipses with different colors represent DEGs belonging to different modules in Figure S8. All correlations with *p* < 0.05 and |PCC| > 0.9 were included. The red edges indicate a positive relationship between nodes while green edges suggest negative relationships.

In lipid cluster (Figure 3A), LysoPC 18:3, LysoPC 18:3 (2n isomer) and LysoPC 16:1 shared similarly positive correlations with several modifying enzymes, including hydrolases, oxidoreductases and transcription factors. The same pattern was found in LysoPC 16:2 (2n isomer) and LysoPG 16:1. However, LysoPC 14:0 was more closely related to a series of transcription factors.

The heatmap of mapped DEGs suggested that most transcripts encoding enzymes in the lysolecithins biosynthesis branch, such as LPLAT (1-acyl-sn-glycerol-3-phosphate acyltransferase), GPAT (glycerol-3-phosphate acyltransferase), DGK (diacylglycerol kinase), PISD (phosphatidylserine decarboxylase) and PLA1/2 (phosphatidylserine sn-1/2 acylhydrolase), showed increased levels after salt treatment. Thus, the altered composition of lysolecithins was attributed to up-regulated transcription levels of structural genes. According to previous research, the expression of *LPLAT* and *PLA2* can be induced by salt stress in *Parachlorella kessleri*, in some way resulting in membrane expansion [27].

After dephosphorylation, 1,2-diacyl-sn-glycerol can directly release fatty acid, for example palmitic acid, which accounts for 47.5% of LBF essential oil [28], can be changed into triacylglycerol with DGAT1 enzymes. It is worth noting that fatty acid linked xanthophyll, known as carotenoid esterification, is common in fruits and vegetables [29]. Importantly, the putative carotenoid esterase found in Arabidopsis (PES1 and PES2) and tomato (PYP1 and PYPLl) were homologous, having diacylglycerol acyltransferase activities [30,31]. We hypothesize that the biosynthesis of palmitic acid or palmitoyl-CoA was increased based on the significantly enhanced content of zeaxanthin dipalmitate in STS4 (Figure S4).

**Figure 4:**
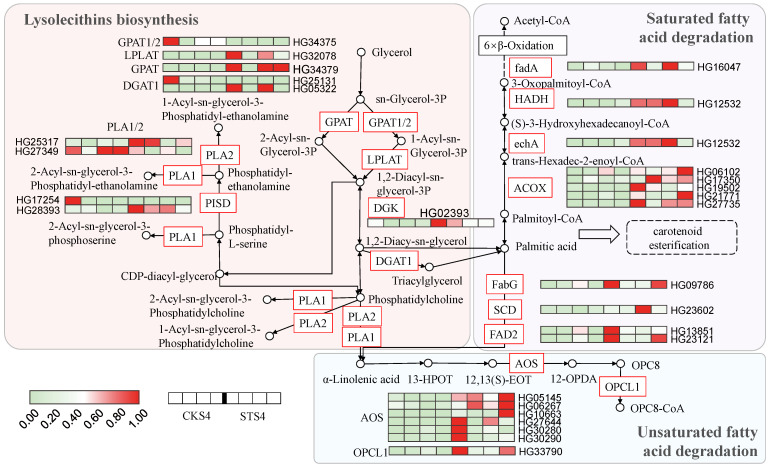
Differentially expressed genes (DEGs) and accumulated metabolites (DAMs) related to lipid metabolism. Rectangle and circle cells indicate the DEG and DAM, respectively. The color key indicates that the transcript levels from low to high are represented from light green to red. CKS4 and STS4 are S4 fruits without and with 300 mM of salt stress treatment. GPAT, glycerol-3-phosphate acyltransferase; LPLAT, lysophospholipid acyltransferase; DGK, diacylglycerol kinase; DGAT1, diacylglycerol O-acyltransferase 1; PLA1/2, phosphatidylserine sn-1/2 acylhydrolase; PISD, phosphatidylserine decarboxylase; FAD2, omega-6 fatty acid desaturase; SCD, stearoyl-CoA desaturase; FabG, 3-oxoacyl-[acyl-carrier protein] reductase; ACOX, acyl-CoA oxidase; echA, enoyl-CoA hydratase; HADH, 3-hydroxyacyl-CoA dehydrogenase; fadA, acetyl-CoA acyltransferase; AOS, hydroperoxide dehydratase; OPCL1, OPC-8:0 CoA ligase 1.

However, the salt stress also led to a higher flux of fatty acid degradation, for both saturated and unsaturated fatty acid. On one hand, palmitic acid can split into acyl-CoA through *β*-oxidation along with NADH and FADH2 electron transporters. Both DEGs mapped in saturated fatty acid degradation pathways have higher expression levels under salt treatment, including *fadA* (acetyl-CoA acyltransferase), *HADH* (3-hydroxyacyl-CoA dehydrogenase), *echA* (enoyl-CoA hydratase) and *ACOX* (acyl-CoA oxidase). On the other hand, palmitic acid turns into unsaturated fatty acid under the catalysis of FabG (3-oxoacyl-[acyl-carrier protein] reductase), SCD (stearoyl-CoA desaturase) and FAD2 (omega-6 fatty acid desaturase) and subsequently splits into short chains. As shown in Figure 4, the expression of *FabG*, *SCD*, *FAD2*, *AOS* (hydroperoxide dehydratase) and *OPCL1* (OPC-8:0 CoA ligase 1) responded to salt stress induction. Unsaturated fatty acid degradation has been reported to be involved in a less fluid plasma membrane that reduces toxic ion permeability in various plants [32].

Generally, salt stress promoted the biosynthesis of phospholipid and fatty acid as well as the degradation of saturated and unsaturated fatty acids. The alteration of lipid composition induced by salt stress may benefit the membrane fluidity and permeability, which enhances the capacity of plant tolerance to salt stress.

### 3.6. Flavonoid and Phenylpropanoid Pathways

In the flavonoid cluster, guaijaverin had an exclusively similar trend with a suite of modifying enzymes, including hydrolases, oxidoreductases, transcription factors and phosphatases (Figure 3B). While pinobanksin, naringenin and naringenin chalcone were highly related to almost the same genes, other flavonoid glycosides were interconnected with DEGs, forming a complex network (Figure 3B).

**Figure 5:**
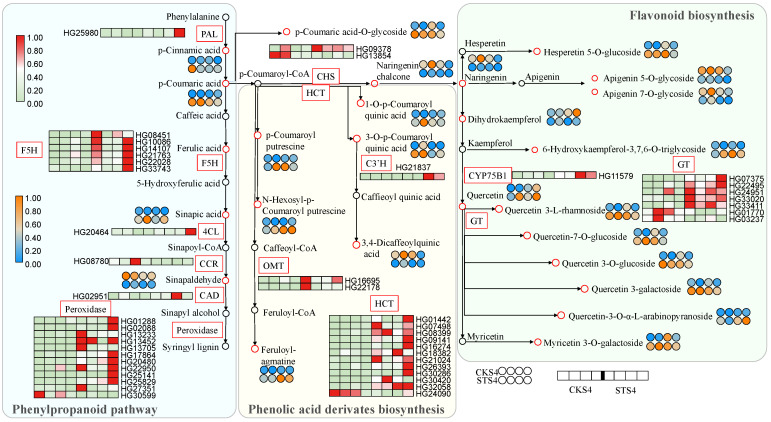
Differentially expressed genes (DEGs) and accumulated metabolites (DAMs) related to phenylpropanoid and flavonoid pathways. Rectangle and circle cells indicate the DEG and DAM, respectively. The color key indicates that the transcript levels from low to high are represented from light green to red, and that the metabolite content from low to high are represented from blue to orange. CKS4 and STS4 are S4 fruits without and with 300 mM of salt treatment. PAL, phenylalanine ammonia-lyase; F5H, ferulate-5-hydroxylase; 4CL, 4-coumarate-CoA ligase; CCR, cinnamoyl-CoA reductase; CAD, cinnamyl-alcohol dehydrogenase; HCT, shikimate O-hydroxycinnamoyltransferase; C3′H, 5-O-(4-coumaroyl)-D-quinate 3’-monooxygenase; OMT, caffeoyl-CoA O-methyltransferase; CHS, chalcone synthase; GT, glycosyltransferase.

In the phenylpropanoid pathway, out of the five DAMs, including *p*-cinnamic acid, *p*-coumaric acid, ferulic acid, sinapic acid and sinapaldehyde, four were up-regulated in the STS4 fruits. Among their biosynthetic structural genes, *PAL*, *F5H*, *4CL*, *CAD* and *Peroxidase* showed higher expression levels under salt treatment (Figure 5). Additionally, phenolic acid-CoA conjugated quinic acid, producing phenolic acid-quinic acid compounds, such as *p*-coumaroyl quinic acid and caffieoyl quinic acid, and it also conjugated polyamines, producing phenylamine, such as *p*-coumaroyl putrescine and feruloyl-agmatine. Most of these conjugated metabolites are enhanced upon salt treatment, which may be attributed to the up-regulated expression levels of *HCT*, *C3′H* and *OMT* induced by salt stress.

In the flavonoid biosynthetic pathway, the amounts of *CHS* and *F3′H/CYP75B1* transcripts were up-regulated by salt stress. The contents of naringenin chalcone, naringenin and dihydrokaempferol were reduced after treatment. Nevertheless, the accumulation of flavonoid glycosides showed a generally higher level in ST samples, for instance, in hesperetin 5-O-glucoside, 6-hydroxykaempferol-3,7,6-O-triglycoside and a suite of quercetin glycosides, which might be responsible for the decline of flavonoid aglycones. It also suggested that the increased flavonoid glycosides might have contributed to the up-regulated gene expression of *CYP75B1* and *GT*.

Taken together, salt stress promoted the biosynthesis of phenylpropanoid and flavonoid, especially for glycosylated flavonoids (Figure 1 and Figure 5) in LBF. Our findings also agree with the recently reported results on *Solanum nigrum* in which the content of quercetin glycosylated form, quercetin 3-*β*-D-glucoside, was increased by treatment with 150 mM of NaCl [10].

### 3.7. Carotenoid Biosynthetic Pathway

As presented in Figure 1 and Figure S4, salt stress resulted in higher levels of free carotenoids and carotenoid esters accumulated in S4 LBF. However, carotenoid biosynthesis ranked as the top pathway in KEGG enrichment of down-regulated DEGs in STS4 vs. CKS4 (Figure S8B). To explain the contradiction, the transcription level of DEGs involved in the carotenoid pathway was inspected carefully. As shown in Figure 3C, zeaxanthin had all negative correlations with DEGs enclosing it.

**Figure 6:**
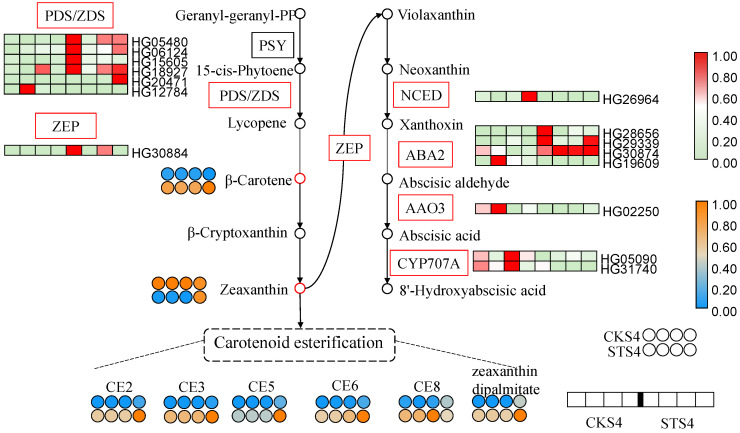
Differentially expressed genes (DEGs) and accumulated metabolites (DAMs) related to carotenoid biosynthesis and metabolism. Rectangle and circle cells indicate the DEG and DAM, respectively. The color key indicates that the transcript levels from low to high are represented from light green to red, and that the metabolite content from low to high are represented from blue to orange. CKS4 and STS4 are S4 fruits without and with 300 mM of salt stress treatment. PSY, phytoene synthase; PDS/ZDS, phytoene/zeta-carotene desaturase; ZEP, zeaxanthin epoxidase; NCED, 9-cis-epoxycarotenoid dioxygenase; ABA2, xanthoxin dehydrogenase; AAO3, abscisic-aldehyde oxidase.

As shown in Figure 6, the structural genes in carotenoid biosynthesis, *PDS/ZDS* and *ZEP*, were mostly activated under salt stress. *ZDS* can reduces zeta-carotene to lycopene, playing a vital role in carotenoid biosynthesis. The Arabidopsis *ZDS* mutant *spc1* showed substantial reduction of carotenoids, enhanced accumulation of superoxide and mosaic cell death [33]. In the carotenoid metabolism branch, the transcript levels of ABA biosynthetic genes, including *ABA2* (HG19609), *AAO3* and *CYP707A*, were decreased in the salt treatment samples to some degree, in turn promoting the accumulation of xanthophylls in STS4 fruit. Thus, salt stress elevated the expression of *PDS*/*ZDS* and repressed the expression of ABA biosynthetic genes, which promoted feedback that led to the accumulation of carotenoids.

Alternatively, as shown in Figure S4, the increased esterified carotenoids in S4 fruit suggested that acyltransferase/esterase plays a critical role in introducing carotenoid flux into ZDP, which facilitates the carotenoid sequestration and accumulation. Recently, a GDSL esterase/lipase was found to catalyze the esterification of lutein in bread wheat [34,35]. It is worth noting that the differentially accumulated carotenoids have a close relationship with several diacylglycerol acyltransferase and GDSL transcripts (Figure 3). Thus, we hypothesize that salt stress promoted carotenoid esterification not only by providing more substrates, both free carotenoids and fatty acids, but also by regulating the expression of esterase genes.

## 4. Conclusions

In conclusion, we found that LBF, at either the green or ripe stage, produced excessive ROS under 300 mM of NaCl treatment. The fruits adopted distinct strategies to deal with the ROS stress triggered by salt stress at different stages. The edible and medicinal tissue of the ripe Goji berry accumulated more antioxidant amino acids and derivatives, as well as flavonoids and carotenoids, under the treatment owing to the enlarging metabolic flux either through up-regulating the expression of structural genes or down-regulating the transcriptional level of degradation genes. Salt stress also stimulated glycosylation of flavonoids and esterification of carotenoids in ripe LBF, which may contribute to the increased capacity of *L. barbarum* plant tolerance to salt stress and further directly and indirectly improve LBF quality. Understanding the accumulation of bioactive components resulting from salt stress modulation is important for crop cultivation and cultivar designation. Our findings show an important potential for improving the nutritional quality of the Goji berry through proper management.

## Data Availability

The transcriptome data that support the findings of this study are available from National Genomics Data Center (https://bigd.big.ac.cn/) (accessed on 27 March 2021) with the access number of CRA004083.

## Supplementary Materials

The following are available online at https://www.mdpi.com/article/10.3390/ijms22094414/s1, Table S1: Metabolites detected in non-target metabolome through UPLC-MS/MS. Table S2: Summary information of RNA-Seq data. Figure S1: Representative TIC plots of UPLC MS/MS data from CKS4 and STS4 samples. STS4 and CKS4 indicate S4 (ripe) fruits with and without 300 mM of salt stress treatment, respectively. Figure S2: Non-targeted metabolome analysis of *L. barbarum* ripe fruits with and without 300 mM of salt treatment. The scores plot (A) and S plot (B) of OPLS-DA analysis of CKS4 and STS4. In the S-plot, red points represent metabolites with VIP > 1. Figure S3: Representatively characteristic absorption spectrum of LBF carotenoid extract at 450nm. Peaks No. 1–3 and 5 are zeaxanthin, *β*-cryptoxanthin and *β*-carotene and lycopene, respectively. Peak No. 4 is a kind of unknown free carotenoid, named FC1. Peaks No. 6–12 and 14 are unidentified carotenoid esters, marked as CE1-7 and CE8, respectively. The highest peak, peak No. 13, is zeaxanthin dipalmitate. Figure S4: The relative content of carotenoids based on peak area in ripe LBF. FC1 is an unknown free carotenoid and CE1-8 are carotenoid esters. All of these compounds are unidentified for lack of standards. STS4 and CKS4 indicate S4 fruits with and without 300 mM of salt stress treatment, respectively. Figure S5: Gene ontology annotation of transcripts derived from *L. barbarum* fruit. Figure S6: Expression levels of transcripts revealed by RNA-Seq and qRT-PCR. The left Y axis represents RPKM values from RNA-Seq, and the right Y axis represents relative expression level from qRT-PCR. CAT, catalase; DGAT, diacylglycerol acyltransferase; SOD, superoxide dismutase; CHS, chalcone synthase; HCT1/2, Shikimate O-hydroxycinnamoyltransferase 1/2; ERF, ethylene response factor, F5H, ferulate-5-hydroxylase; ZEP, zeaxanthin epoxidase; ZEP, zeaxanthin epoxidase; PSY1/2, phytoene synthase 1/2; PDS1, phytoene desaturase; PIF3, phytochrome interacting factor 3; MADS2, MADS-box transcription factor; AAO3, abscisic-aldehyde oxidase. Figure S7: Transcriptomic characterization of *L. barbarum* fruit. (A) Clustering of *L. barbarum* fruit samples based on global gene expression profiles by principal component analysis (PCA). (B) Numbers of up-/down-regulated DEGs in pair-wise comparisons. Figure S8: GO (A) and KEGG (B) enrichment analysis of DEGs. A. GO terms enrichment of DEGs of STS1 versus CKS1 (STS1 vs. CKS1) and STS4 vs. CKS4. B. KEGG enriched pathway of down-regulated and up-regulated DEGs in comparisons of STS4 vs. CKS4. The *x*-axis of all the scatter plots describes the ratio of number of DEGs in the term/pathway compared to the total number of genes mapped in the term/pathway. Dot size represents the number of different genes and the color indicates the *p*-value. CKS1 and STS1 and CKS4 and STS4, are S1 and S4 fruit samples treated without and with 300 mM of salt stress, respectively. Figure S9: The coexpression network of differentially expressed genes (DEGs) and differentially accumulated metabolites (DAMs) in *L. barbarum* fruit. DEGs are represented by ellipses while DAMs are represented by rectangles. All correlations with *p* < 0.05 and |PCC| > 0.9 were included. Red edges indicate positive correlation, while green edges represent negative correlation. AcetylT, acetyltransferase; PrenylT, prenyltransferase; GlycosylT, glycosyltransferase; SAMeT, SAM methyltransferase; AminoT, aminotransferase; CYP450, cytochrome P450; HPX, haem peroxidase; GST, glutathione S-transferase; PCE, PC-esterase; NudH, nucleoside phosphate hydrolase; FAH, fatty acid hydroxylase; *α*/*β* H, *α*/*β* hydrolase; AE, acetylesterase; PhoE, phosphoesterase; GH, glycoside hydrolase; PecE, pectin esterase; ZF, zinc finger domain; AA, amino acid and derivatives; OA, organic acids; Nucls, nucleotide and derivatives.
