# Supplementary figures and images for: Integrative Analysis of Transcriptome and Metabolome Reveals Salt Stress Orchestrating the Accumulation of Specialized Metabolites in Lycium barbarum L. Fruit
Source: Int J Mol Sci. 2021 Apr 23;22(9):4414. doi: 10.3390/ijms22094414 (PMC8122869; doi:10.3390/ijms22094414)

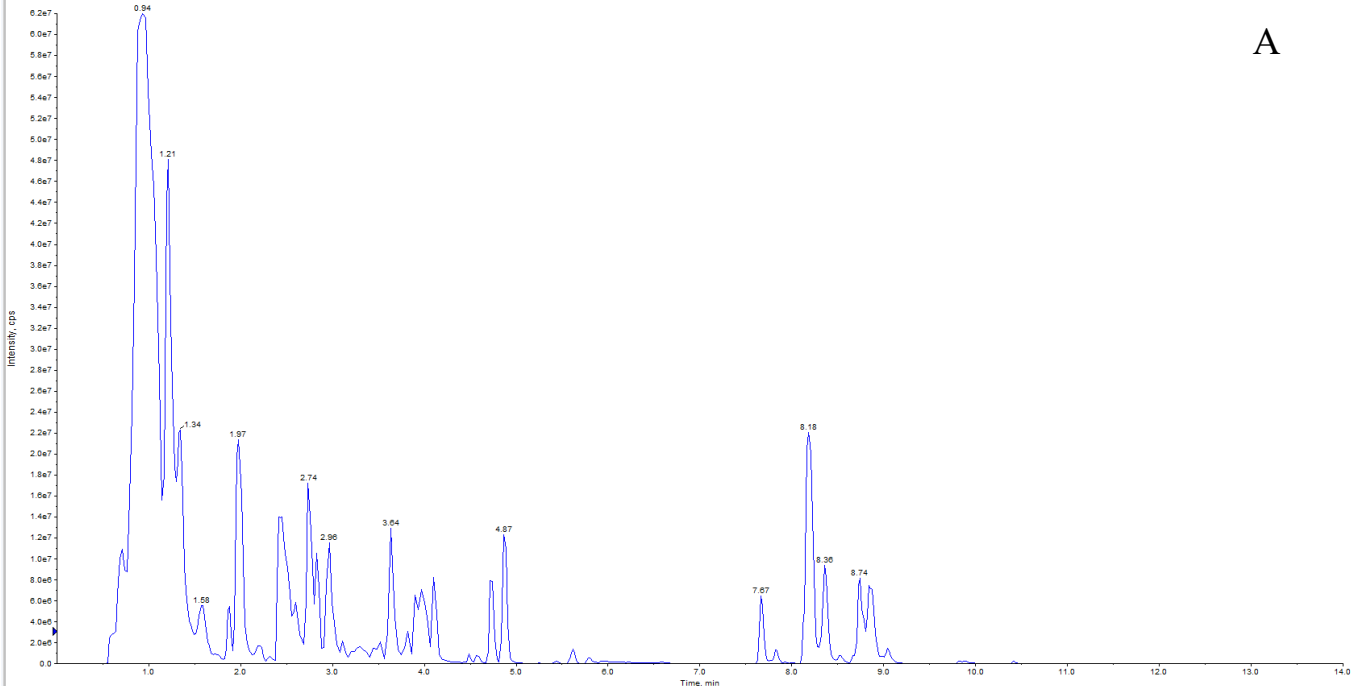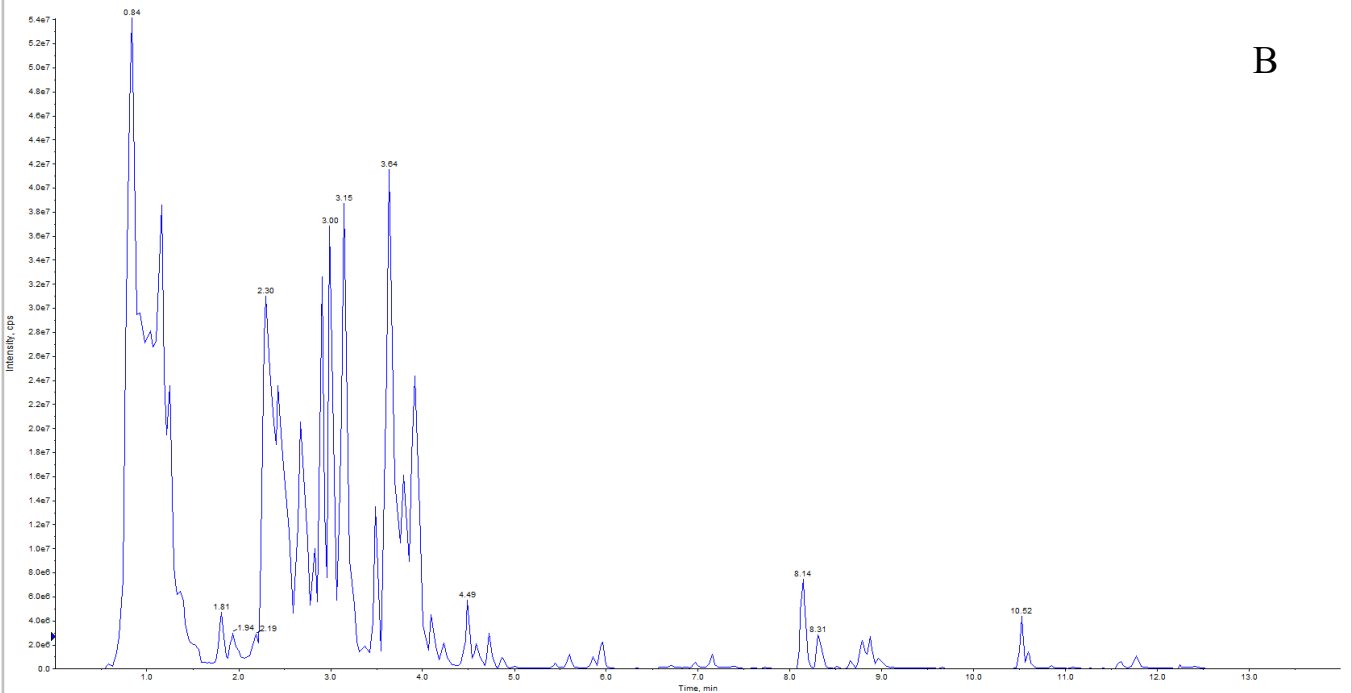

Supplement: Supplementary file 1 [file ijms-22-04414-s001.zip › Supplementary - proof/Figure S1.pdf]

Scores OPLS-DA Plot

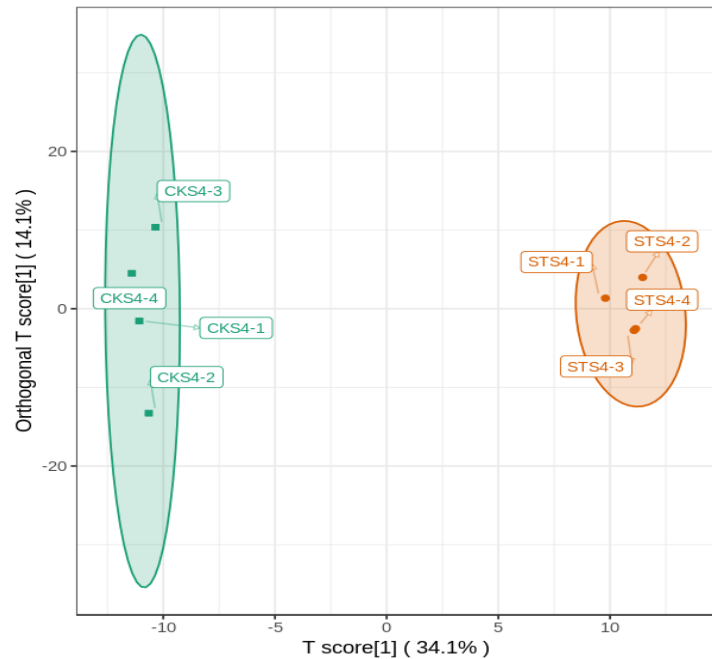

OPLS-DA S-Plot

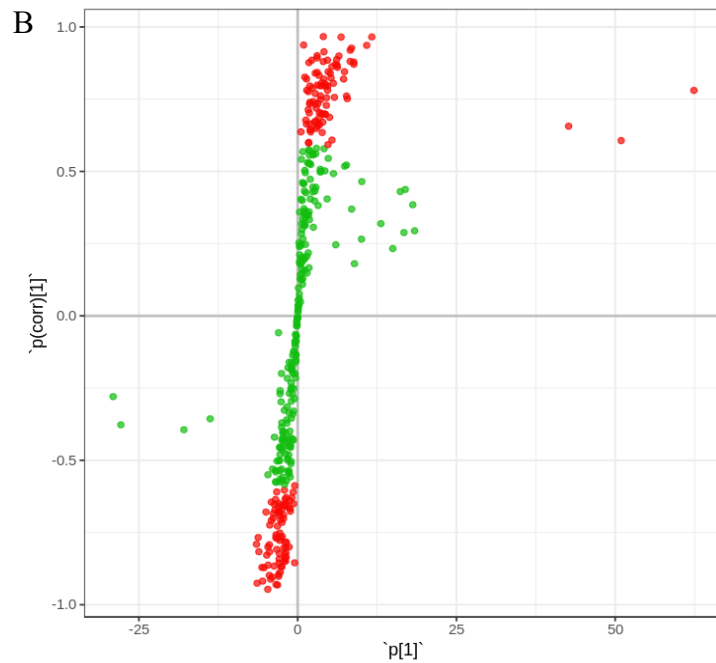

Supplement: Supplementary file 1 [file ijms-22-04414-s001.zip › Supplementary - proof/Figure S2.pdf]

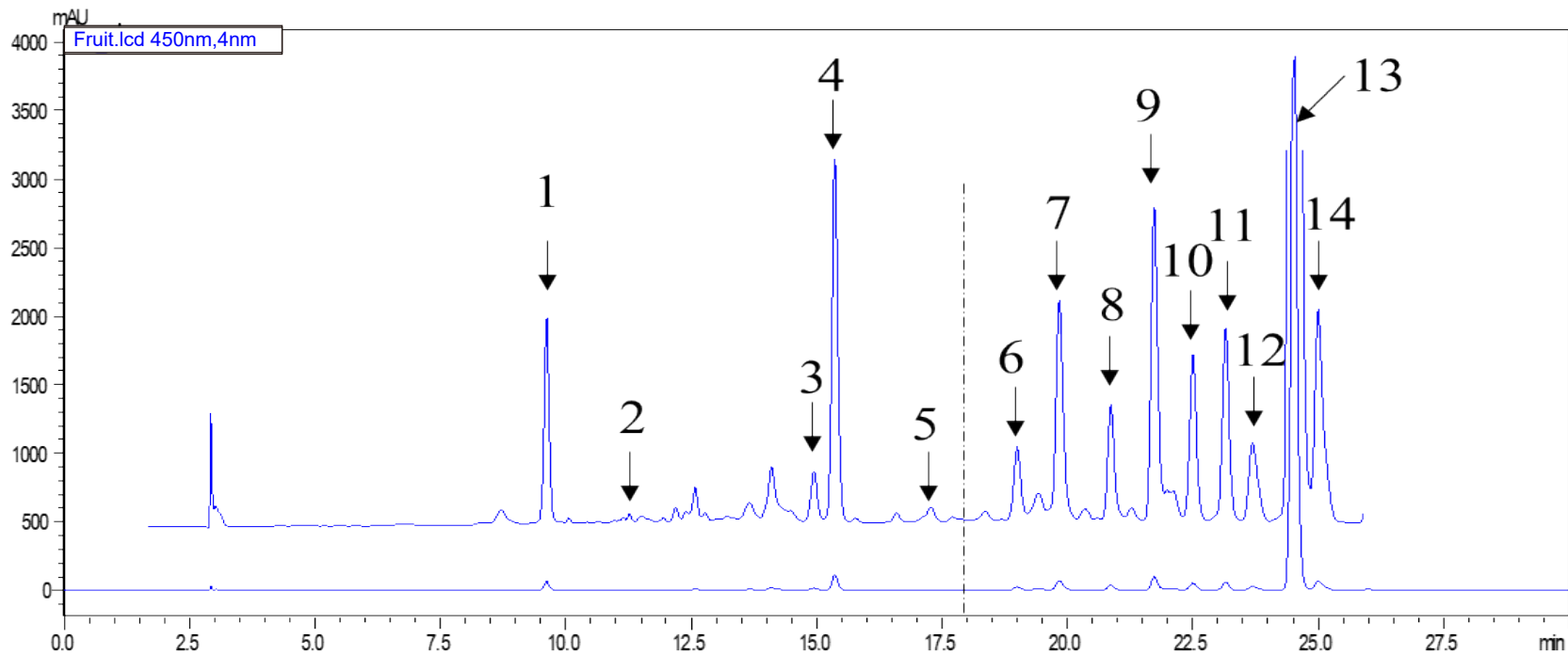

Supplement: Supplementary file 1 [file ijms-22-04414-s001.zip › Supplementary - proof/Figure S3.pdf]

Relative Content

peak area

$5 \times 10^7$   
 $2 \times 10^7$   
 $1.1 \times 10^6$   
 $8 \times 10^5$   
 $5 \times 10^5$   
 $2 \times 10^5$   
0

CKS4  
STS4

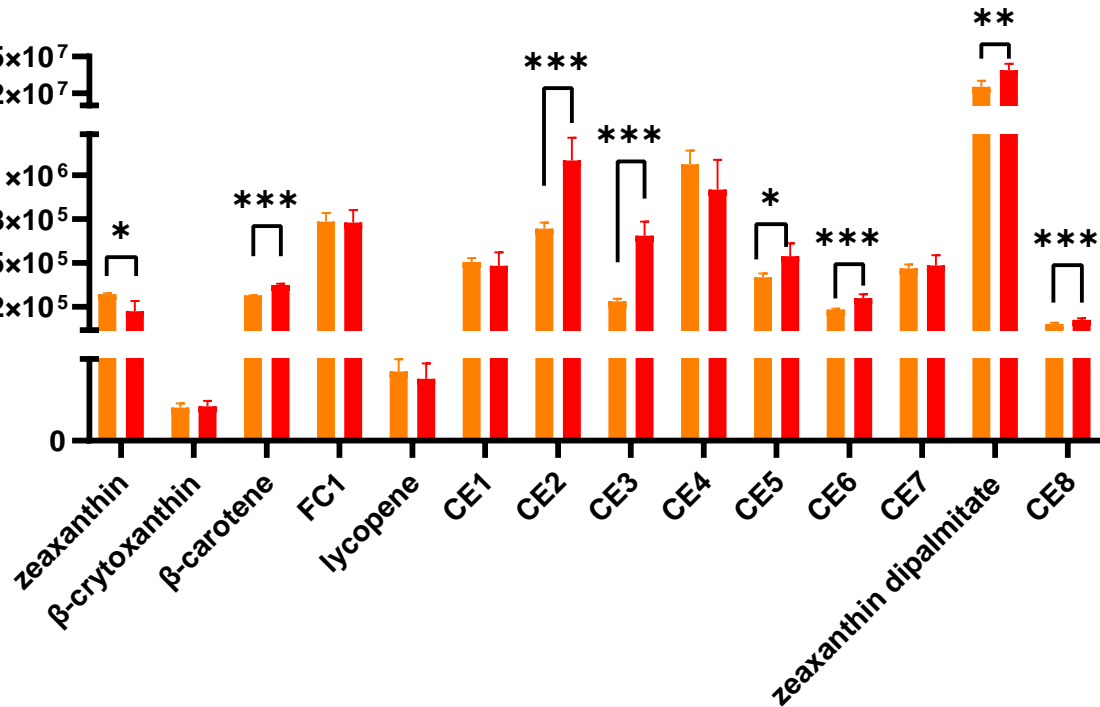

Supplement: Supplementary file 1 [file ijms-22-04414-s001.zip › Supplementary - proof/Figure S4.pdf]

Level2 GO terms

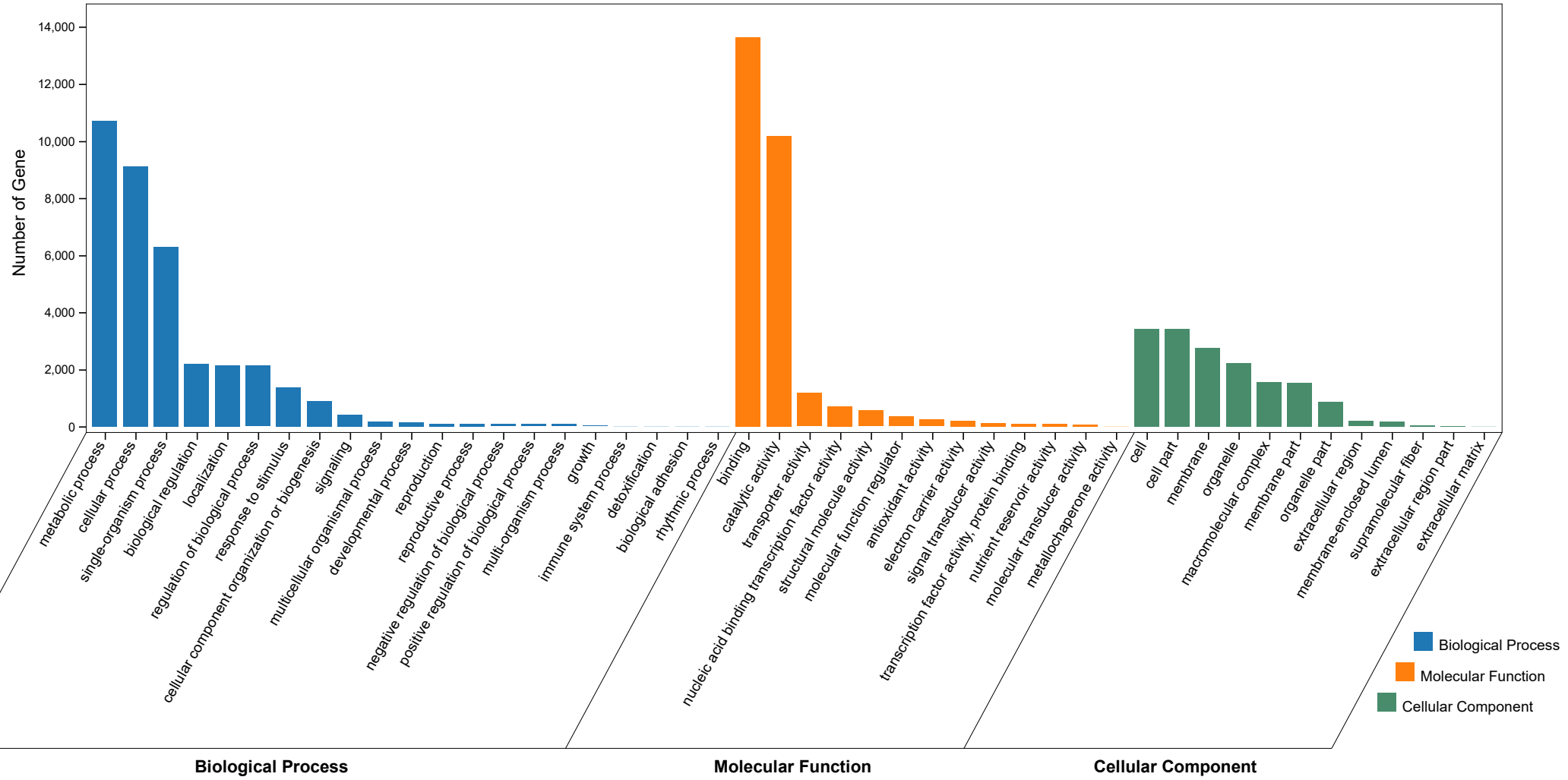

Supplement: Supplementary file 1 [file ijms-22-04414-s001.zip › Supplementary - proof/Figure S5.pdf]

CAT

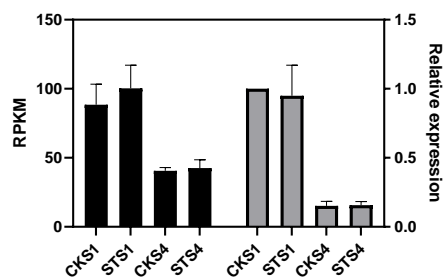

DGAT

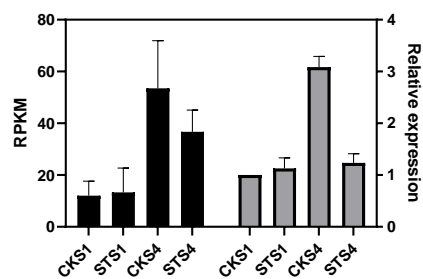

SOD

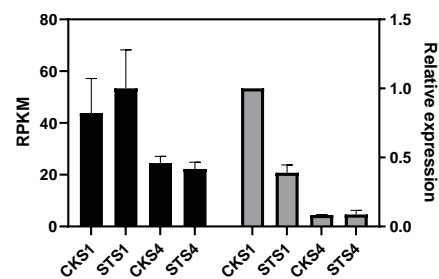

CHS

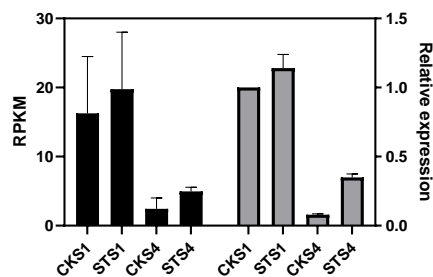

HCT

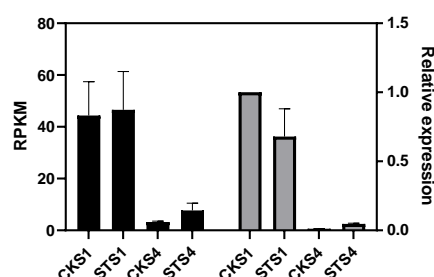

ERF

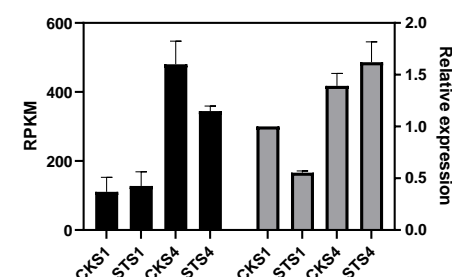

F5H

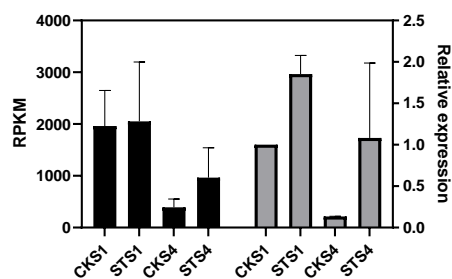

Lipase

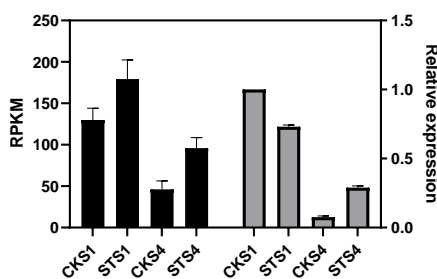

ZEP

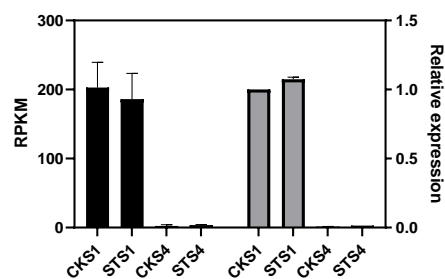

CYP707

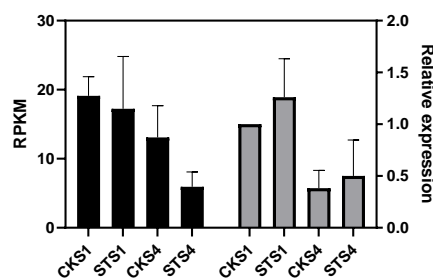

PSY

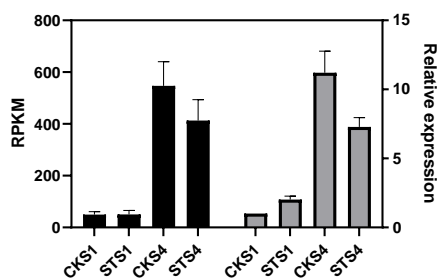

PDS1

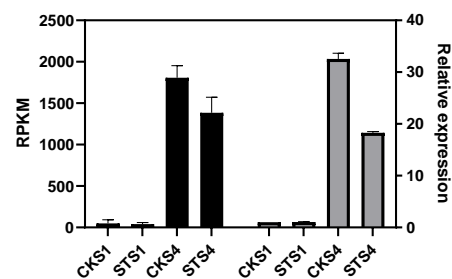

PIF3

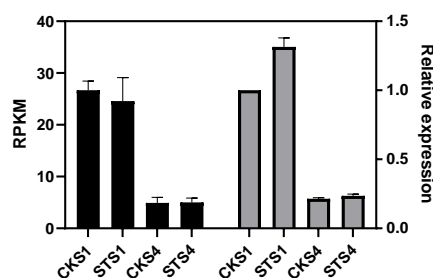

MADS2

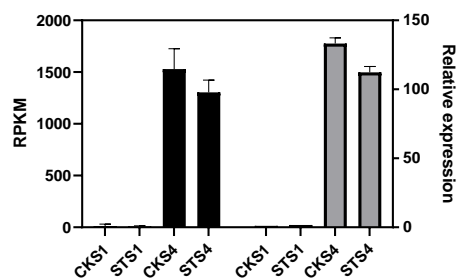

AAO3

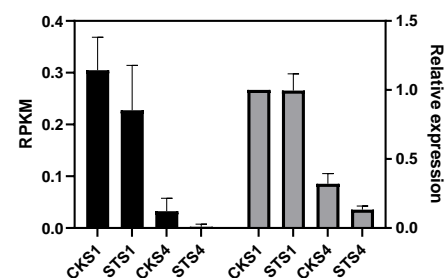

■ RNA-Seq    ■ qRT-PCR

Supplement: Supplementary file 1 [file ijms-22-04414-s001.zip › Supplementary - proof/Figure S6.pdf]

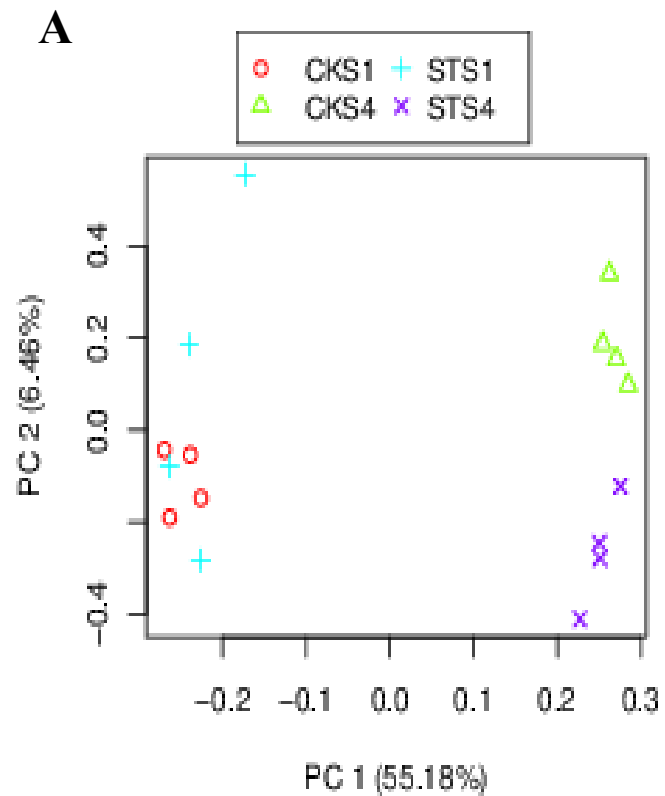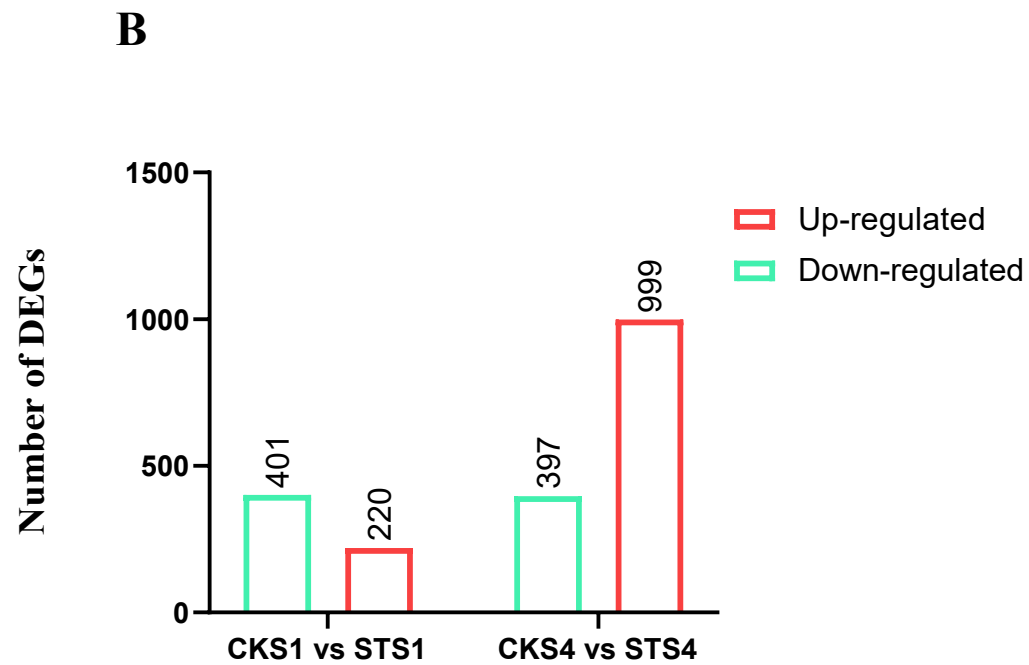

Supplement: Supplementary file 1 [file ijms-22-04414-s001.zip › Supplementary - proof/Figure S7.pdf]

A

STS1 VS CKS1

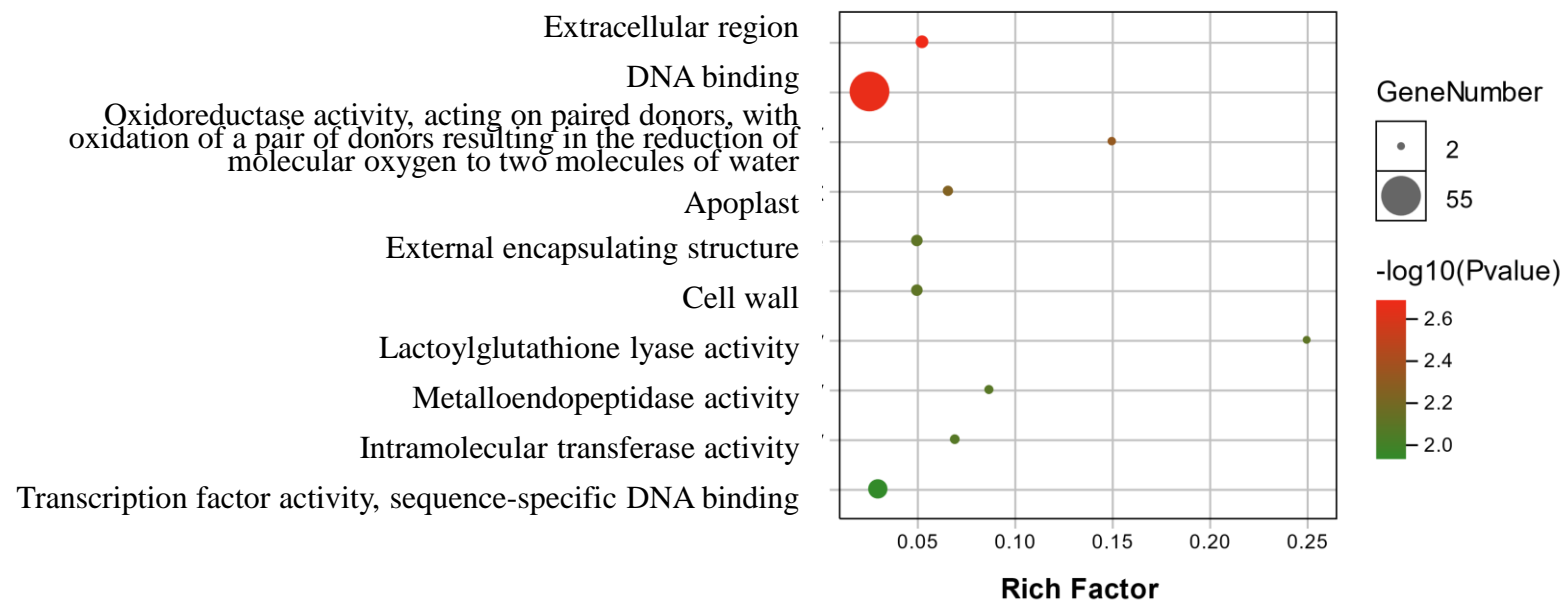

STS4 VS CKS4

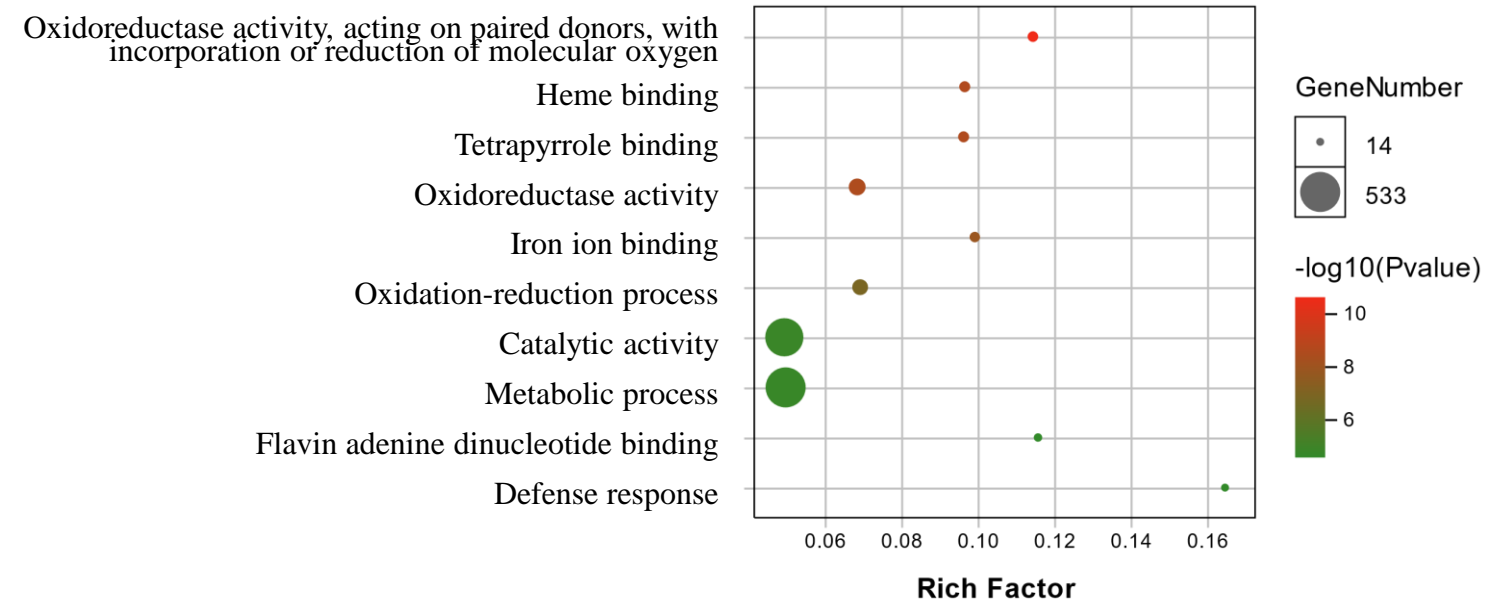

B

STS4 VS CKS4 (Down-regulated)

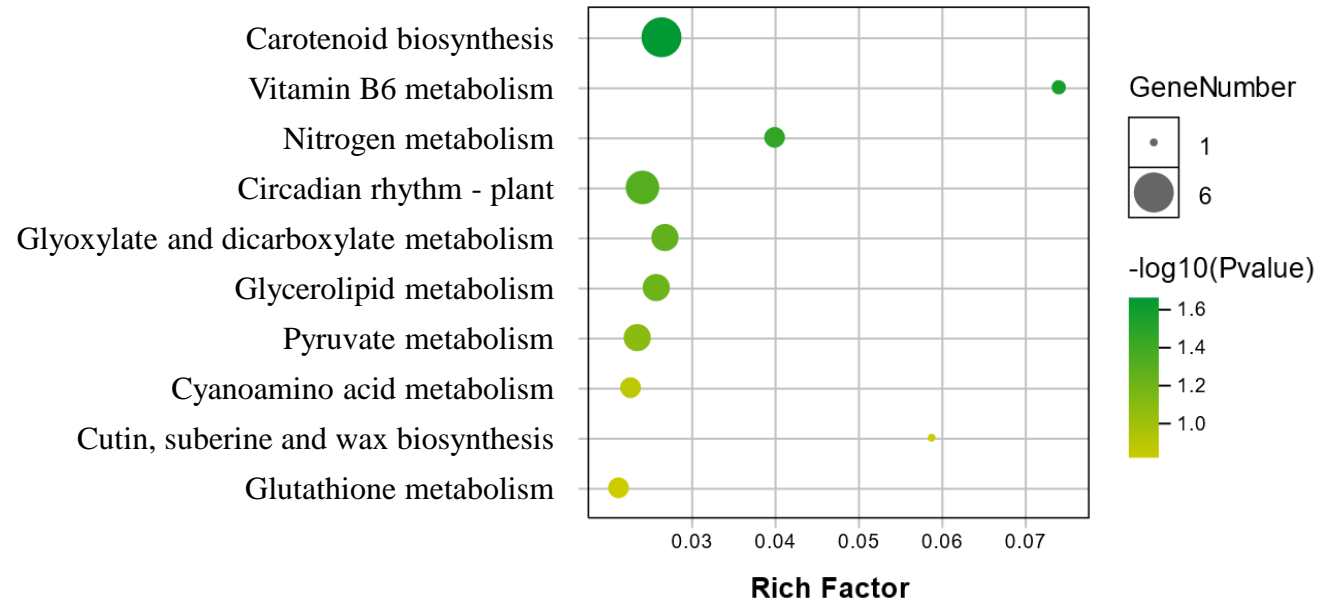

STS4 VS CKS4 (Up-regulated)

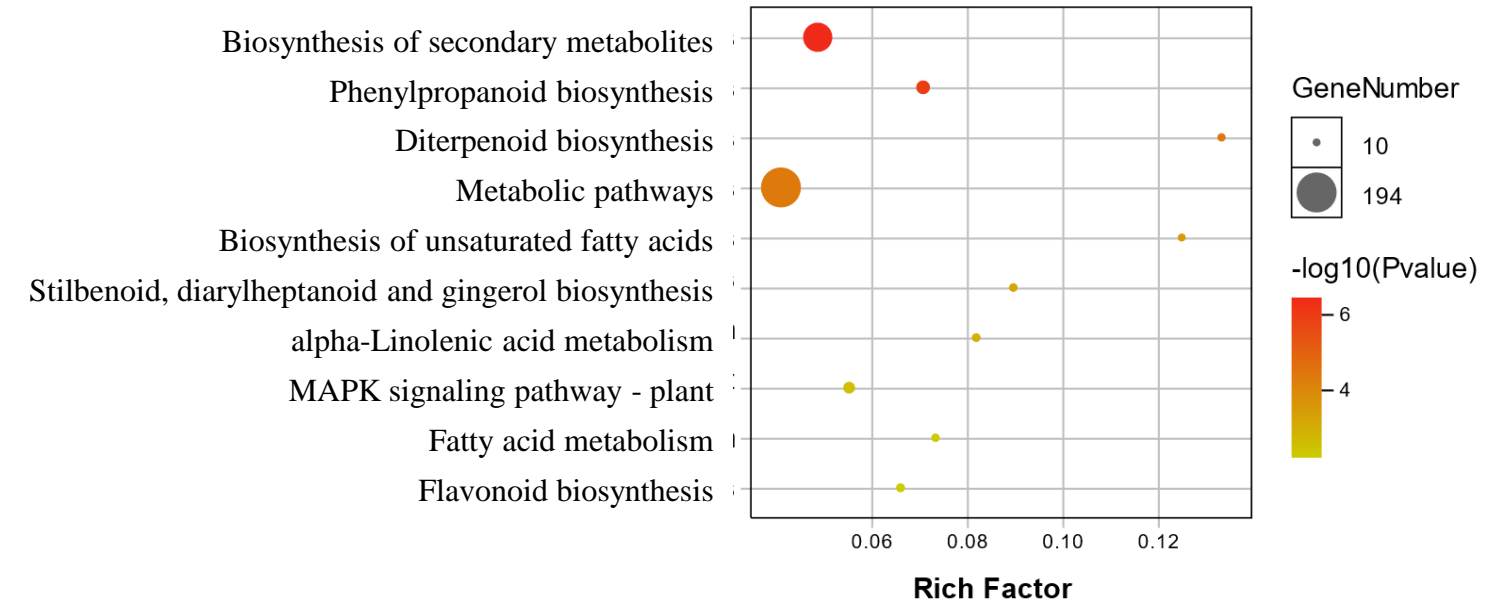

Supplement: Supplementary file 1 [file ijms-22-04414-s001.zip › Supplementary - proof/Figure S8.pdf]

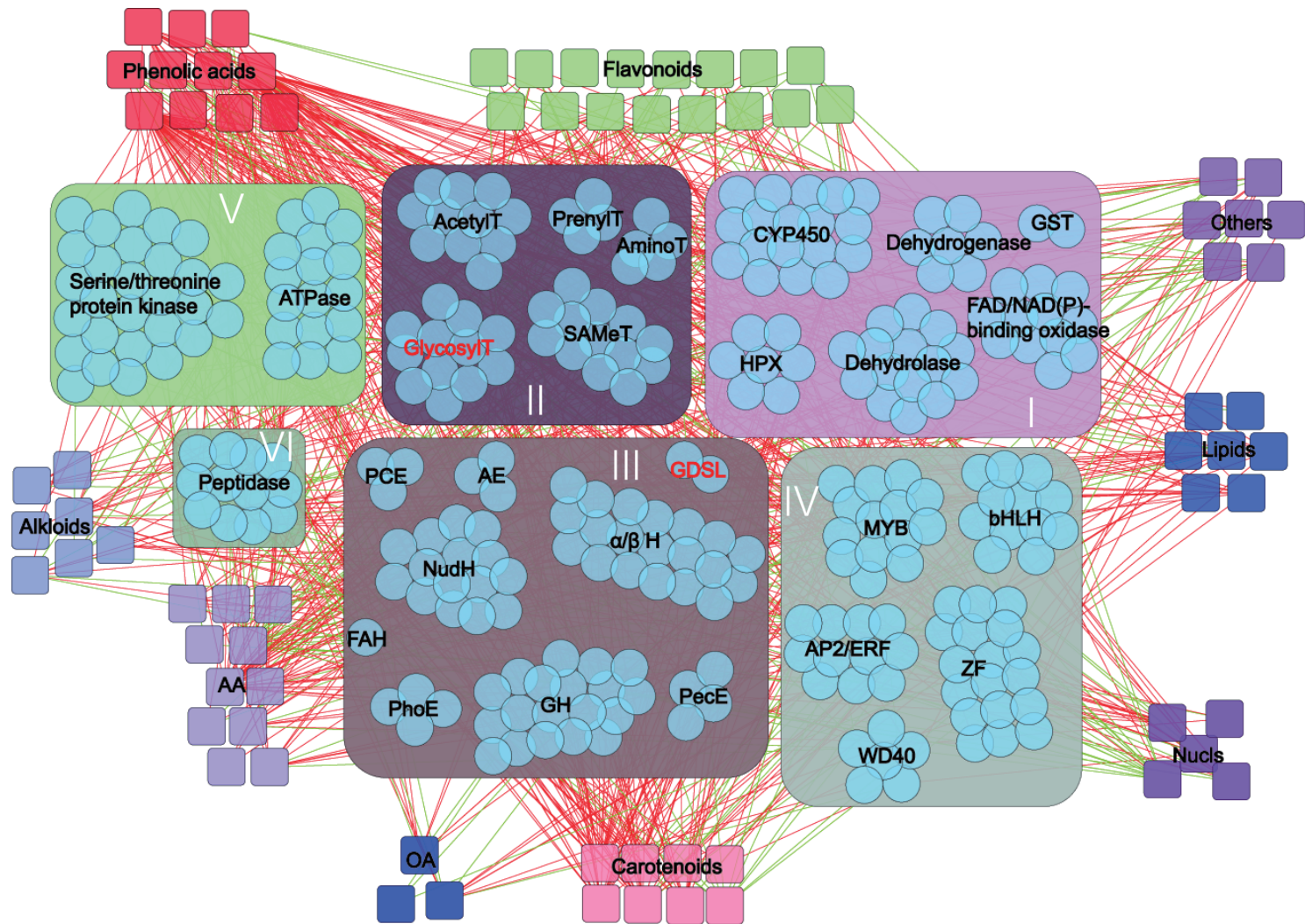

Supplement: Supplementary file 1 [file ijms-22-04414-s001.zip › Supplementary - proof/Figure S9.pdf]
